# Contributions and challenges of cross-national comparative research in migration, ethnicity and health: insights from a preliminary study of maternal health in Germany, Canada and the UK

**DOI:** 10.1186/1471-2458-11-514

**Published:** 2011-06-29

**Authors:** Sarah M Salway, Gina Higginbottom, Birgit Reime, Kuldip K Bharj, Punita Chowbey, Caroline Foster, Jule Friedrich, Kate Gerrish, Zubia Mumtaz, Beverley O'Brien

**Affiliations:** 1 Centre for Health and Social Care Research, Sheffield Hallam University, 32 Collegiate Crescent, Sheffield, S10 2BP, UK; 2 Faculty of Nursing, University of Alberta, University Terrace, 8303-112 Street, Edmonton, T6G 2T4, Canada; 3 Bernhard-Nocht-Institute for Tropical Medicine, Bernhard-Nocht-Str. 74, Hamburg, D-20359, Germany; 4 Leeds Institute of Health and Social Work, School of Healthcare, University of Leeds, Baines Wing, Leeds, LS2 9JT, UK; 5 Op de Elg 52, 22393 Hamburg, Germany; 6 Department of Public Health Sciences, University of Alberta, University Terrace, 8303-112 Street, Edmonton, T6G 2T4, Canada

## Abstract

**Background:**

Public health researchers are increasingly encouraged to establish international collaborations and to undertake cross-national comparative studies. To-date relatively few such studies have addressed migration, ethnicity and health, but their number is growing. While it is clear that divergent approaches to such comparative research are emerging, public health researchers have not so far given considered attention to the opportunities and challenges presented by such work. This paper contributes to this debate by drawing on the experience of a recent study focused on maternal health in Canada, Germany and the UK.

**Discussion:**

The paper highlights various ways in which cross-national comparative research can potentially enhance the rigour and utility of research into migration, ethnicity and health, including by: forcing researchers to engage in both ideological and methodological critical reflexivity; raising awareness of the socially and historically embedded nature of concepts, methods and generated 'knowledge'; increasing appreciation of the need to situate analyses of health within the wider socio-political setting; helping researchers (and research users) to see familiar issues from new perspectives and find innovative solutions; encouraging researchers to move beyond fixed 'groups' and 'categories' to look at processes of identification, inclusion and exclusion; promoting a multi-level analysis of local, national and global influences on migrant/minority health; and enabling conceptual and methodological development through the exchange of ideas and experience between diverse research teams. At the same time, the paper alerts researchers to potential downsides, including: significant challenges to developing conceptual frameworks that are meaningful across contexts; a tendency to reify concepts and essentialise migrant/minority 'groups' in an effort to harmonize across countries; a danger that analyses are superficial, being restricted to independent country descriptions rather than generating integrated insights; difficulties of balancing the need for meaningful findings at country level and more holistic products; and increased logistical complexity and costs.

**Summary:**

In view of these pros and cons, the paper encourages researchers to reflect more on the rationale for, feasibility and likely contribution of proposed cross-national comparative research that engages with migration, ethnicity and health and suggests some principles that could support such reflection.

## Background

Public health researchers are increasingly encouraged to establish international collaborations and undertake cross-national comparative studies. Funding streams such as the European Commission's Framework programmes explicitly require such research designs, while others strongly encourage this approach. Recent investments in the production of harmonized datasets (such as EURO-PERISTAT, [1] and EUROTHINE [2]) and the fielding of international surveys (such as the European Social Survey [3]) also encourage researchers to undertake cross-national research, as do individual and organisational assessments of research quality (such as the Research Excellence Framework in the UK) that reward international partnerships.

The field of migration and ethnic diversity might reasonably be considered an obvious domain for such cross-national comparative research. The majority of high income countries have experienced significant levels of immigration (both planned and enforced) in recent decades with consequent increased ethno-cultural diversity. There is considerable variation, both within and across countries, in how these processes have been problematised and responded to, with divergent opinions expressed as to: the nature of the 'problem'; the goals of integration; appropriate relationships between migrants and their new country; and the obligations of government and wider society to newcomers [4]. There would therefore seem to be great potential for mutual learning and joint problem-solving through collaborative and comparative research in relation to migration and ethnicity. Indeed, a number of important comparative studies focusing on immigration, integration, cohesion, discrimination, (in)equality and related issues have been published in recent years [5,6]. It is perhaps surprising therefore that there has to-date been relatively little cross-national comparative work within the field of ethnicity and health. For instance, our review of the 10 issues of *Ethnicity & Health *including and preceding volume 15(4) published in 2010 revealed that none of the 58 papers published included any such international comparative dimension. Similarly, the 10 most recent issues of *International Journal of Migration, Health & Social Care *dating back from volume 6(1) published in 2010, included just four papers with an international comparative focus out of 43 original articles. Further, where such research has been undertaken it has tended to focus on recent immigrants, rather than more established minorities.

One factor that might discourage public health researchers from taking an international comparative design is the subscription to differentialist conceptualisations of ethnicity. Recent years have witnessed something of a backlash against earlier health-related research which tended to portray ethnic group identities as natural and fixed and to seek explanations for health disadvantage largely in genetic or cultural factors [7-9]. Understandings of ethnic identity that foreground its contingent, contested and fluid nature may sit uneasily with cross-national comparative research. Studies that seek to compare the experiences and outcomes of migrant/minority groups across national settings might be seen to at best offer little in the way of analytical purchase and at worst to privilege genetic or culturalist accounts (since they might implicitly assume an ethnic 'essence' that exists irrespective of time or place).

However, recent work has argued that comparative research can be useful precisely because there is a need to take social context seriously and it allows an exploration of how the significance of ethnic identities varies over time and place [10]. We further suggest that such work can offer other advantages in relation to research that engages with issues of ethnicity, migration and health. Comparative analyses offer the potential for new insights into how the wider socio-political context impacts upon experiences and outcomes of healthcare. More generally, comparative studies provide an ideal opportunity to set aside routine approaches to addressing problems and to engage in critical analyses of current policy and practice [11,12]. Comparative analyses can potentially: highlight the competing priorities operating in different contexts; make visible taken-for-granted assumptions and underlying ideologies; reveal the arbitrariness of particular categorisations and concepts; and suggest innovative solutions.

Nevertheless, studies that include more than one national context and involve researchers based at disparate institutions operating within contrasting academic environments imply significant additional conceptual, methodological and logistical complexity, as well as increased costs, when compared to national studies. Bhopal [13] identifies several factors that can hamper cross-national comparative research on ethnicity and health, including: differing migration histories and policy contexts; differences in the use of ethnic categories and in routine data collection; and divergent perspectives regarding the desirability and need for such research. It is also clear, however, that divergent approaches to undertaking comparative research are emerging in the field of migration, ethnicity and health, raising fundamental questions about how and why such research should be undertaken. Yet, unlike other fields [14,15] public health researchers have not to-date given considered attention to the opportunities and challenges that cross-national comparative studies offer to the field of migration, ethnicity and health. Given the likely growth in such comparative research, we suggest that it is important to debate the value of this work and to seek to identify key issues for reflection. The present paper contributes by drawing on the insights and findings of a recent preliminary study focused on maternal health.

In 2008, an international study group comprising researchers from Germany, Canada and the UK received funding from VolkswagenStiftung to undertake a six month scoping study focused on the maternity experiences and outcomes of immigrant and minority ethnic women in the three contrasting countries. The funding source clearly dictated a focus on Germany, while the UK and Canada were felt to offer the potential for useful comparative analyses given their common commitment to accessibility of public health services and their sustained attention to issues of migration and ethnocultural diversity within both policy and research arenas. A range of activities were undertaken within each of the three countries consisting of: a comprehensive narrative review of literature and policy documents; electronic and telephone consultation with selected experts including practitioners, policy-makers and researchers; and a participatory consultation workshop with service users, practitioners and researchers. These in-country activities resulted in a series of briefing papers which then formed the basis of a two-day symposium at which the project members from the three countries shared findings. The three-country symposium allowed for comparison of: the wider context of migration and ethno-cultural diversity, including concepts and terminologies and potential areas of confusion and contention; issues and challenges to maternity service delivery for migrant and minority populations; and exploration of the similarities and differences between the country settings in terms of the research evidence base and priorities for future knowledge generation. Specific emphasis was placed on examining how migration and consequent ethno-cultural diversity had been problematized and responded to within the respective healthcare systems.

Primarily intended to inform the development of a larger programme of international comparative work (whose aim would be to gain broad theoretical and practical insights into how healthcare systems might promote better maternal health outcomes for migrant and minority women), the objectives of the preliminary work reported on here were to 1) establish a clear and comprehensive conceptual framework informed by, and having pertinence to, the three countries of focus, 2) establish a detailed methodological approach for further investigation, and 3) establish an explicit operational structure that would enable active involvement of policy-makers, practitioners and service users. The present paper reflects on the experiences of the project team in addressing these objectives to identify various challenges and opportunities offered by such international comparative research.

In the sections below we highlight some general issues that would likely be relevant to cross-national comparative public health research irrespective of the exact substantive focus. However, since a number of papers have discussed cross-national comparative health and social research in general terms [16,17], our main focus is the ways in which research focused on ethnicity, migration and health is helped or hindered by an international comparative approach. We aim to alert the reader to issues that require careful reflection and to suggest some principles that may guide future work.

## Discussion

The discussion that follows is organised into three sections following the objectives listed above, namely: identifying a conceptual framework; designing a methodological approach; and establishing an operational structure. Within each section we summarise the key observations from the preliminary studies undertaken, highlighting (i) insights gained from the comparative approach and (ii) challenges raised by exploring the issues cross-nationally.

### 1. Identifying a common, cross-national conceptual framework

In the initial stages of our project, we chose to define 'migrant/minority' women broadly as those women who trace a significant part of their family heritage outside their current country of residence. We recognised the need to explore the ways in which ethnic and migrant statuses are understood across the three countries, rather than impose any rigid conceptualisation or operationalisation from the outset. Our preliminary studies confirmed significant diversity in the language and concepts employed within the three countries in research, policy dialogue and everyday societal discourse that engages with issues of migration and ethnicity. Furthermore, the cross-national comparative work highlighted the way in which our choice of terms and the meaning attached to key concepts is socially and historically situated, as well as the complex and contentious nature of such concepts. We highlight here four areas where particular divergence was apparent, and where this divergence prompted important reflection on current conceptualisations adopted by researchers in the three country teams; their strengths and limitations.

### Terms, categories and labels

We found instances of: the same term being used to mean different things both within and across the country settings; certain terms carrying pejorative connotations in one or more of the countries while being acceptable in the others; and certain terms being commonly used in one setting with no conceptual equivalent existing in the other two countries.

For instance, there was confusion over the terms 'migrant' and 'immigrant'. Researchers in Canada pointed out that whereas 'migrant' tends to carry the connotation of temporary or seasonal movement, 'immigrant', in their context, refers to someone who has moved to the country with the intention of settling permanently and 'landed immigrant' is the official term for someone who has been granted the right to live in Canada permanently. In Germany, the distinction between permanent and temporary migrants to the country is emphasised through the use of the terms *Gastarbeiter *(guest worker) and *Ausländer* (foreigner) or *Aussiedler *(resettler), reflecting different legal statuses and entitlements. In contrast, the UK researchers felt that the terms 'migrant' and 'immigrant' were used fairly interchangeably -perhaps reflecting a less rigid categorisation of people entering the country and the possibility of those on fixed-term work permits deciding to settle permanently and gain citizenship. However, the UK researchers also alerted the team to the negative connotations that the term 'immigrant' can carry, and noted that though the term 'migrant' is sometimes used in public health and epidemiological work to encompass all those born outside the UK, neither 'migrant' nor 'immigrant' would tend to be used for the children or grandchildren of people who had migrated to Britain. Similarly, though the terms 'foreign born' and 'foreign origin' have been used in the UK, they have carried distasteful associations and are uncommon. In contrast, the German team noted that German scholars do not reject the term 'migrant' for the second generation, and cited Schönwälder [18] who argues that the use of this term *(Einwanderer*) is not perceived as othering or excluding in the German context. Nevertheless, some scholars have opted to use the phrase 'individuals with a migration background', in recognition of the problematic nature of the label 'migrant'.

Germany was unique in its use of specific terms to distinguish between migrants of German ethnic background, termed *Aussiedler *(resettler) and migrants of ethnic background other than German, termed *Ausländer *(foreigner). It was also noted that the latter are constructed in policy discourse and the popular media as much more problematic than the former. Despite no conceptual equivalent, research team members in both Canada and the UK did identify collective terms that served to distinguish 'types' of migrant or minority and which could be seen to contribute to the racialisation of particular groups. So, for instance, the term 'visible minority' is used in Canada and the collective 'Black and minority ethnic' or 'BME' is used in the UK, both of which place emphasis on skin colour, and by implication a degree of cultural distance from the majority White population, as well as group together people with very diverse backgrounds and circumstances. Contrary to the UK and Canada, the German team reported that the terms 'race' and 'ethnicity' are rarely used and the concept of ethnic minorities is uncommon in Germany [18]. The German situation is, of course, similar to much of Europe where past abuses have given rise to strong concerns regarding the identification of ethnic identity in official data.

Differences were also evident in the standard practices and specific categories and labels employed by statutory authorities and researchers to identify and enumerate migrant or ethnic 'groups'. So, for instance, in Germany population registers exist and the Federal Institute for Statistics provides regular updates on the 'foreign population' by legal status and the population size by country of citizenship, while ethnicity and language are rarely monitored. In Canada and the UK, population level data on migrants/minorities come primarily from the censuses, though the concepts, questions and categories employed diverge importantly. Aspinall [19] has noted the inclusion in Canadian state data collection of ethnic origin/ancestry (originally defined through the paternal ancestor) as well as 'population group'. Indeed, in the most recent Canadian census in 2006 a range of questions were included relating to migration and ethnicity (for 20% of people who received the long form), asking about 'ethnic or cultural origins of ancestors' as well as country of birth of both parents, citizenship, languages spoken and a question that categorises people into one of the following categories by simply asking 'Is this person...?': White, Chinese, South Asian, Black, Filipino, Latin American, Southeast Asian, Arab, West Asian, Korean, Japanese, Other-specify. In the UK, data on migrant status and ethnic group are collected at census, the latter since 1991, but not ancestral origins. In recent years, use of the 2001 Census ethnic categories has been required in statutory agencies including the health sector, and, though data are far from complete, it is common to find information on service use and outcome by ethnic group. Many large-scale national surveys also gather data on ethnic group. The 2001 census in England asked respondents, 'What is your ethnic group?' and provided five major response options each with sub-categories: White - (British; Irish; Other); Mixed - (White and Black Caribbean; White and Black African; White and Asian; Other); Asian or Asian-British - (Indian; Pakistani; Bangladeshi; Other); Black or Black British (Caribbean; African; Other); Other ethnic group - (Chinese; Other). Clearly, the ethnic categories in use in Canada and the UK differ greatly, with some categories carrying different meanings in the two contexts and some having saliency only in one setting. Furthermore, there has been change over time in both question wording and in response options in both countries.

It is beyond the scope of the current paper to describe comprehensively the diversity of terms and their meanings in use across the three countries. Instead, our objective here was to highlight how our cross-national comparative work vividly illustrated the way in which the concepts and terms relating to migrant/minority populations are neither natural nor neutral entities, but rather are the product and producer of particular forms of social relations. These observations highlighted the importance of: (i) clearly articulating our understandings of the key terms to be used in the research in ways that acknowledged their socio-historical foundations; and (ii) carefully considering the utility and limits of any ethnic or migrant categories employed - aspects that would not ordinarily come under scrutiny in single country work.

In addition, a more challenging issue was raised - *Is it possible or desirable to develop a set of concepts, terms and categories that are relevant across all three countries? *This is a contentious issue. While some researchers advocate working towards standardized instruments and categories for use across diverse settings [13,15], others argue that processes of 'ethnogenesis' are so historically and geographically specific that such harmonization is impossible and unwise [14]. This tension relates to the fundamental epistemological question of how comparative research should steer a course between identifying the similarities across, and the differences between, the settings under investigation [20,21]. Our preference was to follow the lead of researchers who have tried to adopt frameworks that encompass both national-level contextual specificity and universal patterns or trends [16,22]. We therefore adopted an approach that did not seek to impose standard concepts or measures (something we felt was neither conceptually nor operationally feasible) but rather to work with the national peculiarities of our three countries. This meant that our theoretical approach incorporated explicit attention to understanding *processes *of identification and categorisation rather than working with fixed categories as taken-for-granted. At the same time, however, this approach did not preclude from our framework the consideration of over-arching processes that shape the maternity experiences and outcomes of migrant/minority women across the three settings. Such an approach seems particularly appropriate to research on migration, ethnicity and health for at least two reasons. First, there is a danger that the adoption of standardized terminology and simplistic comparisons of fixed migrant/minority categories risks the reification of concepts and essentialisation (whether in genetic or culturalist terms) of migrant/minority populations. Second, this area of work, perhaps more than any, demands that our theoretical frameworks reflect the 'social realities of the postmodern world' [14, p58]; realities in which international migration, ethnic diasporas and transnational identities are juxtaposed with the contextual specificities of particular settings. Our approach therefore sought to work both with and against the established discourses in the three countries to understand the social locations of migrant/minority groups and the implications for maternal health. Such an approach, we felt, offered the potential for important new insights. At the same time, it was recognized that moving beyond accepted concepts and terms may create problems at a country-level. We were conscious of the need to engage with local stakeholders and to generate research products that were meaningful and applicable to these actors; suggesting the importance of employing familiar concepts and terminology. Even the phrase 'migrant/minority women', which we adopted within the project as a shorthand for women who trace a significant part of their family heritage outside their country of residence, would require explanation at the country level. Clearly, the more creative and flexible approach, while potentially fruitful, would undoubtedly necessitate additional effort.

We also recognise that our proposed approach is by no means the norm in health focused research. Indeed, there are a growing number of quantitative 'variable' analyses that compare the health experiences and outcomes of groups of people similarly labelled and categorised across countries [23-26]. While careful attention to the comparability of the outcome measures across national settings is a hallmark of these studies, we suggest that such work would benefit from similar scrutiny of the utility and meaning of the migrant/minority categories employed, particularly if the ambition is to move beyond simple description, to explanation of differences and/or prescriptions for policy and practice.

### Understanding migrant/minority healthcare experiences and outcomes in wider socio-political context

The diversity of concepts and labels in use highlighted above indicated a need for the whole research team to have an appreciation of the historical and present-day patterns of immigration and the policy and societal discourses relating to migration and ethnicity across the three countries. Our preliminary work therefore included the preparation of briefing papers that described these aspects for each country and allowed comparison across the settings. The key issues identified across the three countries showed both similarities and divergence, as summarised below in Table 1:

**Table 1:** 

	*Germany*	*Canada*	*UK*
*Migration patterns*	Post WW2 arrival of displaced/forced migrants from eastern Europe, followed by large numbers of guest workers in 1950s-1970s, followed by asylum seekers and migrants from disintegrating socialist countries in 1990s.	Between 1950s and 1980s immigration predominantly from Europe (UK, Italy) and the US. From 1980s onwards, increasing numbers of arrivals from Asian countries. In the 1990s, immigration rates were at their highest and three quarters of new arrivals were 'visible minorities'.	Significant post WW2 immigration from ex-colonies in South Asia and the Caribbean as well as from Poland and Ukraine, fluctuating over time with changing immigration rules. Fluctuating numbers of asylum seekers since 1990s. Significant European migration since EU expansion, notably from Poland, but often temporary.
Population diversity*	A large and long-established population of migrant background (20% of total population). Numerically, people of Turkish citizenship are by far the largest migrant group. Migrants from non-European countries are gaining increased attention in recent years.	Around 20% of the total population was born outside Canada. The 'Chinese' are identified as the most populous 'visible minority' (25% of whom are Canadian-born).	Latest estimates show 16% of the population belongs to an ethnic group other than 'White British', and 11% were born outside UK. Largest enumerated ethnic groups are 'Indian' and 'Pakistani'. India is most common country of birth outside UK, followed by Poland.
*Policy orientation*	Reluctance to embrace an ethnically diverse identity at policy and societal level [27]. A history of firmly anti-immigration policy orientation. Citizenship based until 2000 on parental heritage rather than country of birth. Persistent resistance to dual citizenship [28]. Recent years, significant tensions and divergent political agendas [29].	Immigrant ancestry and multiculturalism are hallmarks of Canadian identity [30,31]. Successive governments across the political spectrum have encouraged immigration and high levels of naturalisation [32,33]. Points based system for accepting migrants since 1967. More recent policy interpretations of multiculturalism emphasise the importance of attachment to Canada and active citizenship, as opposed to maintenance of cultural distinctiveness [28].	Long recognised itself as an immigrant-receiving and multi-ethnic country [34,35]. Self-perception of a strong legal and policy race relations framework. Critics argue that race equality legislation and policy conflict with that relating to immigration control, citizenship, cohesion, as well as foreign policy [36,37]. Significant backlash against multiculturalism in past decade, though consistent policy concern with diversity and equality (despite limited evidence of progress towards a fairer society) [38].
*Migrant/minority rights and welfare*	Persistent discriminatory treatment of migrants categorised as not ethnic German by state institutions as well as within housing and employment sectors [39]. Low rates of naturalization [28]. Poor socioeconomic indicators among most immigrant groups, particularly Turkish [28]. Some significant recent intervention to recognise and address needs of immigrants [40].	Generally acknowledged success in accommodating diversity [28]. Extensive programmes and resources directed towards 'integration' initiatives [28]. But, veneer of tolerance and celebration of diversity masks structural barriers to economic and social wellbeing of non-European migrant groups who have higher levels of unemployment and lower income than other groups [31].	Introduction of additional requirements for citizenship in 2000s. Relatively low naturalisation rates. Recently characterised as having a 'weak' integration policy [41]. Persistent socioeconomic disadvantage among migrant and minority groups, particularly those categorised as Bangladeshi and Pakistani [42]. Evidence of continued high levels of racial discrimination at institutional and inter-personal levels [28].
*Societal discourses*	Public fears of threat to identity and economic welfare. Significant public suspicion of Muslims [43]. Mass media stereotyping and pathologising of migrants, particularly Muslims [44].	Harsh criticism is levelled at government approaches to multiculturalism that are seen to ignore the hierarchies of power and opportunity that perpetuate poor welfare outcomes for racialised groups [45-47]. Significant concerns about cohesion and Canadian identity expressed by some sections of the general public, particularly among Québécois, though broadly positive public opinion towards immigration [48]. Surveys suggest high levels of racial discrimination in society, particularly among some groups [49]; Racism is increasingly recognised as a critical issue by policy makers [50].	Widespread public concern that immigration levels are too high posing threats to identity and economic opportunities [28]. Media contributing to misinformation and negative stereotyping of migrant/minority communities. Islamophobia a particular problem [51-53].

* Given the divergent categorisation and labelling of migrant and minority groups across the three countries we adopt here the country-specific conventions for describing diversity.

In addition to identifying areas of divergence and commonality that would provide the backdrop for our subsequent investigations, the process of looking across the three national contexts served to highlight a number of more fundamental themes that deserved a greater focus within the conceptual framework for our study.

First, multiple strands of policy and legislation were seen to have relevance to the ways in which migrant/minority groups - their characteristics, needs and entitlements - are constructed and responded to by the state and other actors; strands which may conflict and undermine, or alternatively support and reinforce. It would therefore be important for us to look beyond the health sector to understand how wider state intervention shapes the healthcare experiences and outcomes of migrant/minority people, including how this varies across 'types' of migrant/minority as categorised by state institutions. Second, the language of policy discourse is frequently imprecise and open to multiple interpretations, so that how policy is forged in the practice of health organisations and professionals - what Lipsky has termed 'street level bureaucracy' [54] - requires careful scrutiny. Our conceptual framework therefore needed to incorporate attention to these processes of policy interpretation and translation. Third, the significant inter-play between public opinion, the media and government action must be appreciated. Wider societal discourses, frequently (mis)informed and fuelled by the media, impact importantly upon the wellbeing of migrant/minority groups via: (i) the daily stress of living with a racialised identity (which often involves direct experience of discriminatory behaviour), (ii) the influence they have on government responses in the form of policy and services, and (iii) the attitudes and behaviours of health professionals who operate within this wider societal context. Our review work suggested that, even where there are very positive strands within government policy, migrant/minority experiences are frequently characterised by social and economic barriers to achieving wellbeing. These observations suggested that rather than adopting the tendency to classify our countries according to some explicit or implicit hierarchy related to how well they served the maternity needs of migrant/minority women, it would be more fruitful to explore the qualitative variation across the countries. Looking for what is positive and negative in the different settings and understanding why this is so might then enable us to synthesise elements of good practice and to translate lessons across contexts [15].

### The significance of migration histories and local factors

A more specific insight that emerged prominently during our cross-national comparative work was the particular significance of migration histories for both individuals and collectives. The migration experiences of particular immigrant groups at particular points in time are shaped by both historical antecedents and prevailing social and economic circumstances, with important implications for their subsequent entitlements and opportunities within the receiving country. For instance, Staniewicz [55] highlighted the way in which the reluctant reception of Polish immigrants to the UK in recent years has contrasted sharply with their more positive post-war experiences. While a focus on migrant status and its implications for health experiences and outcomes has been quite prominent in past research in Germany and Canada [56-58], this is less apparent in UK health research [59]. Indeed, the cross-national comparative work alerted the UK research team to the evident gulf between health research that foregrounds migration (and which tends to focus on recent immigrants, refugees and asylum seekers) and that which foregrounds ethnicity (which tends to focus on established post-colonial communities) and the potential benefits of integrating these strands of work. Closely related to this insight, was the observation that there can be significant within-country variation in patterns of migration and the associated experiences of migrant groups, shaped by local economic, social and historical specificities, as well as the particular responses of local and regional state institutions and other actors. Again, the importance of considering diversity within the nation-state was immediately apparent to the researchers in Canada and Germany, where federal political systems result in very obvious policy divergence between regions. These observations encouraged the UK researchers to reflect on patterns and causes of internal diversity in migrant/minority identifications, experiences and outcomes, and how these might be considered within the research design. Furthermore, the comparative approach raises the more fundamental question of whether the nation-state is the most useful and meaningful unit of analysis [15,20]. A focus on migrant/minority groups suggests the importance of exploring flows and networks rather than constructing imaginary communities that are geographically bounded and stable. Nonetheless, there is clearly a need to recognise the nation-state as a meaningful policy and legal environment as well as to balance the demand for country-relevant research findings with broader knowledge that can contribute to our understanding of global issues.

### The place of indigenous minorities

A further issue raised related to whether and how the experience of indigenous populations should be incorporated into research focused on migrant/minority women. While the Canadian researchers argued that indigenous peoples were neither migrants nor likely to self-identify as 'minorities', they stressed that academic, policy and public discourse in relation to migration and diversity is shaped by the legacy of colonialism and attempted cultural genocide. While this Canadian historical context was clearly distinct from that of the UK or Germany, it nevertheless prompted important questions regarding whether and how our research should engage with the UK's traveller and gypsy communities (and the failure to-date of most official sources to identify and enumerate these people - though the 2011 census will include a category) and Germany's differential treatment of *Ausländer *and *Aussiedler *migrant populations. Again, this suggested the importance of constructing a study that was not bound by a focus on particular migrant or ethnic *categories*, but that could explore broader issues of identification, categorisation, inclusion, and exclusion in a more flexible way.

### 2. Designing a viable and appropriate methodological approach

Turning to the design of a methodology that would be feasible and appropriate to the three settings, the cross-national nature of our study again created challenges as well as opportunities.

### Identifying meaningful research questions

There were significant differences across our three countries in terms of both the volume of existing research in the field of migrant/minority maternal health and the prevailing policy and practice context of maternity services. This presented challenges in terms of identifying a set of research questions which, if answered, would be meaningful for each of the country contexts, as well as provide opportunities for broader comparative analysis. We were aware of past criticisms that much comparative research fails to go beyond descriptive accounts of the individual countries included to produce deeper interpretations and genuine integration of scholarship [15,17,20]. In addition, however, we were concerned that our findings should be pertinent to national policy-makers and practitioners, lending themselves to translational activities, rather than being of purely theoretical or academic interest.

Our cross-country preliminary work identified common issues facing migrant/minority women and maternity services across the three countries despite their divergence in social, policy and service constellation, including, among others: ineffective cross-language and cross-cultural communication; discrimination, stereotyping and insensitivity at provider and program levels; and a failure to recognize and respond to the complex issues within some migrant women's lives (such as trauma, isolation, mobility, poverty). Such transcending issues were therefore taken as the starting point to develop a broad set of research questions that had general applicability. At the same time, however, the contrasting knowledge bases and service contexts demanded that we also generate country-specific research questions to guide data generation and analysis.

In addition, the review of existing evidence across the settings forced the research team to reflect on (i) the nature, source and implications of biases in the existing data sources and evidence base, and (ii) our responsibilities as researchers to consider not just the rigour of the present study, but our contribution to the wider evidence base and the extent to which it serves the needs of all sections of the population.

### Working with divergent data sources

A further complicating factor was the significant differences across our three countries in the availability and accessibility of data sources that might lend themselves to secondary analysis. For instance, while both the UK and Canada had recently conducted surveys of women's maternity experiences which included samples (albeit relatively small) of migrant/minority women [60,61], no such comparable German data were available. There were also important differences in terms of available administrative data on maternity service use with the UK having ethnicity data and the other countries only country of birth. Furthermore, while the UK and Canada had quite a large amount of qualitative evidence relating to maternal health experiences of migrant/minority women, this type of evidence was virtually non-existent in Germany. These divergent situations meant it was simply inappropriate to employ identical research designs in the three settings. Instead, we adopted a design that involved new qualitative data generation in Germany and meta-synthesis of existing qualitative evidence combined with secondary analysis of survey data in the UK and Canada. In contrast, some elements of our study were designed to be conducted more-or-less similarly across the three countries and this was more feasible where new data collection was warranted for all three countries, for instance an interpretive analysis of policy documents and key informant interviews with policy-makers. Clearly, then, the cross-national nature of our study added significant complexity to the methods of data generation we had to employ.

### Integrating data analysis and interpretation across the country settings

The approach to research design described above meant that we could ensure the viability and relevance of analyses in all three countries. It did, however, raise the issue of how to avoid the project developing into three parallel studies operating independently rather than an integrated whole yielding more than the sum of its parts. As discussed above, our conceptual work clearly indicated a comparative approach intended to generate insights that extended beyond the country-level contexts to more over-arching processes. The issue of how data should be integrated across divergent settings in cross-national work is widely acknowledged to be challenging [20,22], but there is little in the way of coherent guidance on how to go about this process in practice. As described above, we were faced with the possibility of generating data from highly divergent socio-political settings using different methodological tools. How then should we design and justify our comparative analytical method?

Here it was helpful to consider how existing models of (i) integrating data in mixed methods studies, and (ii) approaches to cross-national comparison within social science, might inform our approach. In relation to mixed methods work, Mason's [62] notion of 'Constructing multi-dimensional explanations' is useful. In contrast to 'triangulation', where the aim of employing different methods is to achieve more accurate measurement and consequently a more valid representation of the phenomenon under investigation, the multi-dimensional approach views different data collection methods as offering alternative perspectives on the social phenomenon of interest. Taking this approach, the complexity, uncertainty and potential contradictions of our cross-national comparative data can be seen positively, offering the possibility of asking distinctive but inter-related questions about migrant/minority maternity experiences. The aim of such an approach would not be integration of the data into one coherent whole, but rather an understanding of the migrant/minority experience as multidimensional. Similarly, Wrede et al.'s [16] notion of 'decentred comparative research', in which there is a conscious effort to ensure both reflexivity and local sensitivity in analysis is helpful. As discussed above, research into migration, ethnicity and health, we believe, demands an in-depth understanding of the socio-cultural, economic and political context. Both these orientations to data analysis and integration view the complexity and 'messiness' of the data generated through such a holistic approach as beneficial rather than problematic.

Nevertheless, a whole host of practical and procedural issues arise in terms of how an international team should actually work with the datasets generated to conduct analyses and generate findings. Furthermore, this type of integration is challenging both for the researchers who may struggle to extract meaning from large volumes of 'untamed' data and for research users for whom the nuance, complexity and uncertainty of resulting research products may be difficult to incorporate into decision-making.

### Incorporating a critically reflexive approach to research practice

A final methodological issue worth noting related to the degree of critical commentary on ethical and scientific rigour in researching ethnicity that was evident across the settings. Thus, while the UK researchers were actively involved in debates regarding whether and how health researchers should engage in research related to minority ethnic populations and the potential for such work to do more harm than good [63,64], this was not so evident for the other country teams. Interestingly in Canada, while there is a well-developed body of literature relating to the ethics of researching with indigenous populations [65], this has yet to be expanded to address migrant/minority groups in any detail. In Germany, too, this area is relatively under-explored. As such, this was an area where it was felt the UK research tradition could usefully enhance the work of the Canadian and German research teams. At the same time, however, we were alerted to the dangers of imposing Anglocentric thinking in other contexts, a tendency that has been highlighted by other commentators on comparative research [15,22] and, as noted above, identified several areas of learning for British researchers.

### 3. Establishing an effective operational structure

In common with many complex international projects involving several partners, a number of differences across the three countries presented operational challenges to effective working. Livingstone [20] has highlighted some of the personal and organisational challenges of cross-national working and Wrede et al. [16] offers useful insights into teams making the most of international collaborative work; we do not reiterate these themes here. However, two issues are worth discussing because of their relation to racialised hierarchies within academia and the generation of knowledge about migrant/minority people: (i) the composition of research teams; and (ii) the involvement of members of the public and service users in research.

### Composition of research teams

Though notions of 'insider' and 'outsider' are complex [66], the social identity of the researcher is a fundamental element in the generation of knowledge about migrant/minority people [67,68]. Furthermore, the extent to which people of migrant/minority background gain opportunities to become researchers is an ethical and political issue. Indeed, recent principles for social research highlight the need to support the careers of minority researchers [69]. Given these concerns, it was important to consider the differential make-up of the research teams and the extent to which there was a pool of potential researchers from diverse migrant/minority backgrounds available in the three countries. In this respect, the UK and Canada appeared to be at an advantage compared to Germany. Thus, while both the UK and Canadian teams included several researchers of migrant/minority identity who were multilingual, this was less so for Germany. That said, researchers from all three countries were conscious of the persistent need to enhance opportunities for minority researchers in their own academic environments, though progress seemed more likely to be achieved in Canada - with its stronger emphasis on the development of neophyte researchers - than in the UK or Germany.

### Service user involvement and participatory research

The involvement of service users and members of the public in health research is a well-established element of research design in the UK. While not without critics (and consisting of a variety of approaches in practice), researchers are expected to adopt strategies that facilitate the involvement of users within the research process and these are commonly subject to scrutiny within research funding and ethical approval processes. User participation in health research is supported by large numbers of third sector organisations as well as a network of state-funded organisations - so-called LINks - set up to promote and support the involvement of local people in the commissioning of health and social care services, and the national-level body, INVOLVE, that has an explicit remit to promote the empowerment of the public to become involved in research [70]. The importance of such involvement is particularly emphasised within the field of migration, ethnicity and health research, with many researchers and consumer organisations arguing that user participation is a fundamental principle of good research in this area [71,72]. In Canada too, there is widespread expectation of service user involvement in healthcare and a well-established research tradition of user involvement with state funding encouraging so-called 'collaborative or engaged scholarship' through the provision of grants for community-university partnerships [73,74]. As in the UK, such approaches have been used widely with community groups representing marginalised populations, particularly indigenous populations [75].

As such, our Canadian and UK researchers had the advantage of well-established networks and past experience of engaging service users and community members in their research; a significant contrast to the German situation where such involvement is much less well developed and few structures exist to support inclusive or participatory research designs. These divergent research contexts were evident in the differential make-up of participants to the three in-country workshops held as part of the project. In the UK and Canada representatives of service user organisations as well as migrant/minority women who had recently experienced maternity services were recruited to the workshops and Project Advisory Groups relatively easily. However in Germany, the lack of pre-existing structures and short time frame made it difficult to identify migrant/minority women willing to engage with the study in this way and other strategies were adopted, including a series of individual interviews, to gain insights from these important stakeholders.

While the differential research contexts prompted debate and offered the potential for learning across the teams, it also added complexity. While the UK and Canadian work would be importantly shaped by the perspectives and priorities of migrant/minority women themselves, such engagement would be much less in Germany. There was no easy solution to this problem, but it did suggest the need for careful and ongoing reflection and documentation of the ways in which such user involvement shaped the generation, interpretation and presentation of data. Furthermore, it raised the question of whether and how migrant/minority service users participating in the UK and Canadian parts of the study should or could be involved in the generation of knowledge relating to Germany and the broader integration of data across the countries alongside members of the research team.

Clearly, the extent to which these aspects of operational structure are considered problematic relates fundamentally to one's epistemological and political stance. For those researchers who argue in favour of user involvement and diversity of research teams both in terms of (i) generating more authentic and useful findings, and (ii) the democratisation of the research process, a cross-national design that cannot ensure these elements, is cause for concern. Indeed, some researchers suggest we should be wary of research accounts that have not been adequately informed by the experiential knowledge of those who are the subject of the research [76].

## Summary

The discussion above has illustrated a number of ways in which our cross-national comparative approach presented both opportunities and significant challenges to researching migrant/minority maternal health.

On the positive side, it would appear that cross-national comparative research can enhance the ethical and scientific rigour of research into migration, ethnicity and health by:

• forcing researchers to engage in both ideological and methodological critical reflexivity;

• increasing awareness that the concepts and methods employed by researchers and the 'knowledge' so generated are intimately bound up with social and historical relations and do not simply reflect an external 'reality';

• engendering an appreciation of the need to situate analyses of health within the wider socio-political setting;

• helping researchers (and research users) to see familiar issues from new perspectives and thereby encouraging innovative solutions;

• encouraging researchers to frame research in ways that move beyond fixed 'groups' and 'categories' to look at processes of identification, inclusion and exclusion;

• encouraging a multi-level analysis that explores local, national and global influences on migrant/minority health; and

• encouraging conceptual and methodological development through the exchange of ideas and experience between diverse research teams.

However, it should also be recognised that cross-national comparative research in migration, ethnicity and health can have downsides, including:

• a tendency to reify concepts and essentialise migrant/minority 'groups' in an effort to harmonize across countries;

• a danger that analyses are superficial, being restricted to independent country descriptions rather than generating integrated insights;

• difficulties of balancing the need for meaningful findings at country level and more holistic products; and

• increased logistical complexity and costs.

The present paper has not sought to advocate one particular approach. Indeed, many questions remain unanswered in our own minds as to the most appropriate way to design and conduct such comparative work. Nevertheless, it is clear that to-date public health researchers have devoted more effort to justifying their research topics than to clearly articulating why and how they intend to adopt a comparative research design. We therefore offer here a number of principles that may help to enhance the usefulness of such research.

### Framing the research: purpose and intended products

We suggest that researchers should:

• Carefully consider why they are adopting a cross-national comparative design, what particular type of comparative analysis is intended, and what can and can not be learned from the proposed research.

• Undertake cross-national comparative work where it offers significant additional analytical purchase, and not merely because of funding imperatives; and clearly articulate the analytical opportunities that would not be achieved through a single country design.

• Think carefully about how research questions are framed to ensure that research does not inadvertently promote or reinforce essentialist or pathologising accounts of migrant/minority people.

• Clearly articulate the relationship between themselves and the commissioners and other stakeholders in the research and how this may shape, and perhaps compromise, the research process.

### Country and team selection

We suggest that researchers should:

• Select countries not out of convenience or in response to funding imperatives, but so as to ensure the research questions can be addressed adequately. Reflect on the degree of similarity or contrast that may be helpful.

• Give thought to whether the nation-state is the most appropriate unit of analysis and whether the research design can usefully incorporate analyses and sub- and supra-national level.

• Reflect carefully on their own ideological and epistemological position in relation to researching migrant/minority health and the degree of compatibility with other members of the proposed international research team.

### Conceptualising and operationalising migrant/minority 'groups'

We suggest that researchers should:

• Give adequate attention to understanding the socio-cultural, economic and political contexts in which migrant/minority people's health experiences and outcomes are situated across the countries of focus.

• Carefully consider the utility and limitations of proposed approaches to conceptualising and operationalising migrant/minority identities and 'groups'. In particular, consider whether the adoption of fixed, statutory categories may reinforce hierarchical power.

• Carefully consider the pros and cons of creating harmonized datasets and of drawing direct comparisons between similarly labelled 'groups' in different countries.

Recent reviews of cross-national comparative research have commonly concluded that the understanding gained is often disappointing given the resources invested [15,20]. While such research offers great potential to public health researchers who are keen to contribute to better health outcomes for migrant/minority populations, our experience suggests the need for much greater reflection on how such research should be designed and conducted. Preliminary studies of the type we undertook here would seem to be invaluable in order to assess the feasibility and likely contribution of proposed cross-national comparative work.

## Declaration of competing interests

The authors declare that they have no competing interests.

## Authors' contributions

SS was PI on the project, produced the first draft and finalised the manuscript.

GH and BR were country leads for the project, contributed to design and execution of the work, and provided critical input to the manuscript.

KB, PC, CF, JF, KG, ZM and BO'B were all team members on the project contributing to the generation and interpretation of the data. All provided critical input to the manuscript.

All authors read and approved the final manuscript.

## Pre-publication history

The pre-publication history for this paper can be accessed here:

http://www.biomedcentral.com/1471-2458/11/514/prepub
